# Differences in the MRI Signature and ADC Values of Diffuse Midline Gliomas with H3 K27M Mutation Compared to Midline Glioblastomas

**DOI:** 10.3390/cancers14061397

**Published:** 2022-03-09

**Authors:** Peter Raab, Rouzbeh Banan, Arash Akbarian, Majid Esmaeilzadeh, Madjid Samii, Amir Samii, Helmut Bertalanffy, Ulrich Lehmann, Joachim K. Krauss, Heinrich Lanfermann, Christian Hartmann, Roland Brüning

**Affiliations:** 1 Institute of Diagnostic and Interventional Neuroradiology, Hannover Medical School, 30625 Hannover, Germany; lanfermann.heinrich@mh-hannover.de; 2 Department of Neuropathology, University of Heidelberg, 69120 Heidelberg, Germany; rouzbeh.banan@med.uni-heidelberg.de; 3 Department of Neuroradiology, INI-Hannover, 30625 Hannover, Germany; akbarian@ini-hannover.de; 4 Department of Neurosurgery, Hannover Medical School, 30625 Hannover, Germany; esmaeilzadeh.majid@mh-hannover.de (M.E.); krauss.joachim@mh-hannover.de (J.K.K.); 5 Department of Neurosurgery, INI-Hannover, 30625 Hannover, Germany; samii.office3@ini-hannover.de (M.S.); a.samii@ini-hannover.de (A.S.); bertalanffy@ini-hannover.de (H.B.); 6 Institute of Pathology, Hannover Medical School, 30625 Hannover, Germany; lehmann.ulrich@mh-hannover.de (U.L.); hartmann.christian@mh-hannover.de (C.H.); 7 Department of Radiology and Neuroradiology, Asklepios Klinik Barmbek, 22307 Hamburg, Germany; r.bruening@asklepios.com

**Keywords:** H3F3A, K27M, diffuse midline glioma H3 K27M-mutant, diffuse midline glioma H3 K27-altered, glioblastoma H3 K27M-wildtype

## Abstract

**Simple Summary:**

Diffuse midline glioma is a rare distinct brain tumor entity introduced by the recent WHO (Lyon, France) brain tumor classification. MR imaging is important for detection as well as characterization of these midline tumors, which are located within the brainstem, since neurosurgical tissue sampling in these locations can be challenging. We therefore searched for possible differences in the MRI pattern between diffuse midline gliomas and midline glioblastomas, both regarded as grade IV entities.

**Abstract:**

We conducted a two-center retrospective survey on standard MRI features including apparent diffusion coefficient mapping (ADC) of diffuse midline gliomas H3 K27M-mutant (DMG) compared to midline glioblastomas H3 K27M-wildtype (midGBM-H3wt). We identified 39 intracranial DMG and 18 midGBM-H3wt tumors. Samples were microscopically re-evaluated for microvascular proliferations and necrosis. Image analysis focused on location, peritumoral edema, degree of contrast enhancement and DWI features. Within DMG, MRI features between tumors with or without histomorphological GBM features were compared. DMG occurred in 15/39 samples from the thalamus (38%), in 23/39 samples from the brainstem (59%) and in 1/39 tumors involving primarily the cerebellum (2%). Edema was present in 3/39 DMG cases (8%) versus 78% in the control (midGBM-H3wt) group (*p* < 0.001). Contrast enhancement at the tumor rim was detected in 17/39 DMG (44%) versus 67% in control (*p* = 0.155), and necrosis in 24/39 (62%) versus 89% in control (*p* = 0.060). Strong contrast enhancement was observed in 15/39 DMG (38%) versus 56% in control (*p* = 0.262). Apparent diffusion coefficient (ADC) histogram analysis showed significantly higher skewness and kurtosis values in the DMG group compared to the controls (*p* = 0.0016/*p* = 0.002). Minimum relative ADC (rADC) values, as well as the 10th and 25th rADC-percentiles, were lower in DMGs with GBM features within the DMG group (*p* < 0.001/*p* = 0.012/*p* = 0.027). In conclusion, DMG cases exhibited markedly less edema than midGBM-H3wt, even if histomorphological malignancy was present. Histologically malignant DMGs and midGBM-H3wt more often displayed strong enhancement, as well as rim enhancement, than DMGs without histomorphological malignancy. DMGs showed higher skewness and kurtosis values on ADC-histogram analysis compared to midGBM-H3wt. Lower minimum rADC values in DMGs indicated malignant histomorphological features, likely representing a more complex tissue microstructure.

## 1. Introduction

Initially, diffuse midline gliomas WHO grade IV, H3 K27M-mutant (DMG), were defined in the 4th revised Edition of the 2016 WHO Classification of Brain Tumors [1] as predominantly astrocytic differentiated gliomas with H3F3A, HIST1H3B or HIST1H3C mutations. Since such mutations were also found in other brain tumors, such as gangliogliomas and ependymomas, cIMPACT-NOW Update 2 clarified the definition of DMG by adding the criterion of diffuse infiltration [2]. In the 5th edition of the 2021 WHO classification, these tumors are now referred to as diffuse midline glioma WHO CNS grade 4, H3 K27-altered. Pontine DMGs are predominantly found in children, yet thalamic and spinal DMGs are more frequently observed in younger adults [3]. While many DMGs histopathologically resemble glioblastomas due to a malignant cellular pattern (DMG-GBMpos) harboring microvascular proliferations (MVP) and/or necrosis, a significant number of these tumors present with the histopathological appearance of a low-grade astrocytoma (DMG-GBMneg). However, because these DMG-GBMneg patients are not prognostically different from DMG-GBMpos patients [4,5,6], these tumors were assigned to WHO grade IV [1] or WHO CNS grade 4 [7] regardless of their appearance.

Due to the localization of malignant diffuse gliomas of the midline, tumor resection is often not possible. Stereotactic biopsy, however, has become a standard procedure available in specialized centers [8,9,10,11]. In general, obtaining a biopsy is also recommended in children; however, this is still debated [9]. Although biopsies or even resection can be performed, histopathological and molecular pathological classification may not be available at the time of diagnosis, which initially then relies on neuroradiological means. This raises the question of the extent to which neuroradiological criteria alone can be used to differentiate DMGs from other entities such as WHO grade IV IDH wild-type glioblastomas of the midline. Recently, the analysis of conventional MRI features of adult glioma molecular subtypes [12,13], as well as the radiogenomic analysis of diffuse intrinsic pontine gliomas [14,15], were published. Moreover, the inclusion of diffusion-weighted imaging data has been announced to add value to the MRI-based prediction of genetic characteristics of gliomas [13], and a histogram analysis of diffusion data was shown to be helpful in the differentiation of posterior fossa tumors [16,17].

In a previous study from a singular neurosurgical institution, we analyzed the MRI signature of 24 DMGs and compared these with a series of 19 glioblastomas WHO grade IV, H3 K27M wildtype, of midline structures (midGBM-H3wt) [18]. We have now expanded this series, adding patients from a second institution, including the evaluation of the diffusion weighted imaging/apparent diffusion coefficient (DWI/ADC) pattern of our cases and controls as an additional criterion. We also compared the MRI signature of DMG with typical histopathological malignancy criteria (DMG-GBMpos) with DMG that presented histopathologically as WHO grade II or III tumors (DMG-GBMneg), thereby investigating the feasibility of discerning DMG from midGBM-H3wt, as well as recognizing DMG subtypes by using MRI features.

## 2. Materials and Methods

Tumor samples from patients of two participating neurosurgical centers, where they underwent neurosurgical treatment with either resection or biopsy between 2005 and 2021, were retrospectively evaluated. The study was approved by the local ethics committee and the patients consented to the scientific use of their medical data/specimens.

The main inclusion criteria was the diagnosis of a diffusely infiltrating glioma of the midline, i.e., located in the thalamus, mesencephalon, pons, medulla oblongata and cerebellum. The tumors had to be neuropathologically identified as either DMG (carrying an H3 mutation) or midGBM-H3wt (a GBM without an H3-mutation). Patients had to be untreated and the pre-therapy MR images had to be available; missing DWI/ADC maps were not an exclusion criterion. Patient characteristics and data regarding cortisone application at the time of or up to 14 days before MR imaging had to be available from patient records. Gliomas of the spinal cord were excluded.

Following the retrospective identification of suitable patients, the stained sections of formalin-fixed paraffin-embedded tissue blocks were retrieved from the neuropathological archive and re-evaluated by two experienced neuropathologists (RBa, CH) according to the definition criteria of the revised 4th edition of the 2016 WHO classification from the year 2016 [1], under consideration of cIMPACT-NOW Update 2 [2]. The slightly different definition criteria of the 5th edition of the 2021 WHO classification regarding DMG and glioblastoma were not taken into account [7]. Geographical or palisading necrosis and MVP were decisively recorded as parameters and defined as criteria for the diagnosis of glioblastoma in addition to diffuse infiltration. Subsequently, DNA was extracted, and H3F3A (H3-3A) status was determined by pyrosequencing. If an H3F3A wild-type status was observed, the tumors were additionally sequenced with respect to HIST1H3B (H3C2) mutations. Details of the molecular pathological analysis can be found elsewhere [19].

### 2.1. MR Imaging and Analysis

Tumor imaging was performed either on a 1.5T or 3T MRI scanner using unenhanced T1- and T2-weighted (w) sequences as well as at least one contrast-enhanced T1w-sequence (ce-T1w); if possible, diffusion weighted imaging and apparent diffusion coefficient (ADC) analysis was performed. Three readers with neuroradiological experience for at least 7 years (RBr, AA, PR) independently judged the MRI data while blinded to each other’s results. The following aspects were scored (adapted from Banan et al. [18]): (1) location (thalamus, brainstem, vermis, cerebellum), (2) absence or presence of intratumoral necrosis, (3) degree of contrast enhancement in a four-step grading scheme of the intensity (strong, intermediate, low or absent), (4) presence of peritumoral edema, (5) presence of mass effect, (6) presence of rim enhancement and (7) occurrence of multifocal tumor manifestations. If a disagreement of the readers was noted, a consensus reading was performed in order to reach a final decision (based on majority).

On ADC maps, 3D regions of interest (ROIs) covering the whole tumor were drawn on solid tumor components in comparison with the T2w-images and ce-T1w-images. Signs of necrosis or hemorrhage (based on T2w-images or available susceptibility weighted images) were excluded from these ROIs. Mean and minimum (min), as well as maximum (max), ADC values were recorded, using the Visage 7.1 PACS software (VISAGE Imaging, Berlin, Germany). For each patient, a 2D ROI at the level of the basal ganglia was used as a within-patient reference value, resulting in relative ADC values (rADC [10^−3^ * mm^2^/s]). By using intraindividual relative ADC values, we controlled for the different DWI sequences available in this retrospective analysis coming from different scanners. Additionally, a histogram-based ADC-texture analysis of the solid tumor tissue was performed using the freely available software package FireVoxel (build 369, https://firevoxel.org/, 7 January 2022) [20]. 3D ROIs were placed as described above together with 3D ROIs of the basal ganglia, with values calculated by the FireVoxel software for ADC percentile values (ADC (10), ADC (25), ADC (75), ADC (90)) and the statistical measurements skewness and kurtosis, as well as entropy. ADC percentile values were referenced to the individual basal ganglia, resulting in relative ADC percentile data (rADC (xx)).

### 2.2. Statistics

SPSS v25 (IBM Corp., Armonk, NY, USA) was used for the statistical analyses. The *p*-values listed are considered significant if *p* < 0.05 (used for all tests) and are calculated 2-sided. Fisher’s exact test was used to analyze group differences in relation to variables of the study, and the *t*-test was used for correlation analyses concerning age and rADC values/ADC-histogram analysis results. The Shapiro–Wilk test was used for the estimation of data-normality distribution as well as data variance within the groups. ROC-/AUC-analysis was performed for significant diffusion data.

## 3. Results

### 3.1. DMG Group

**Figure 1:**
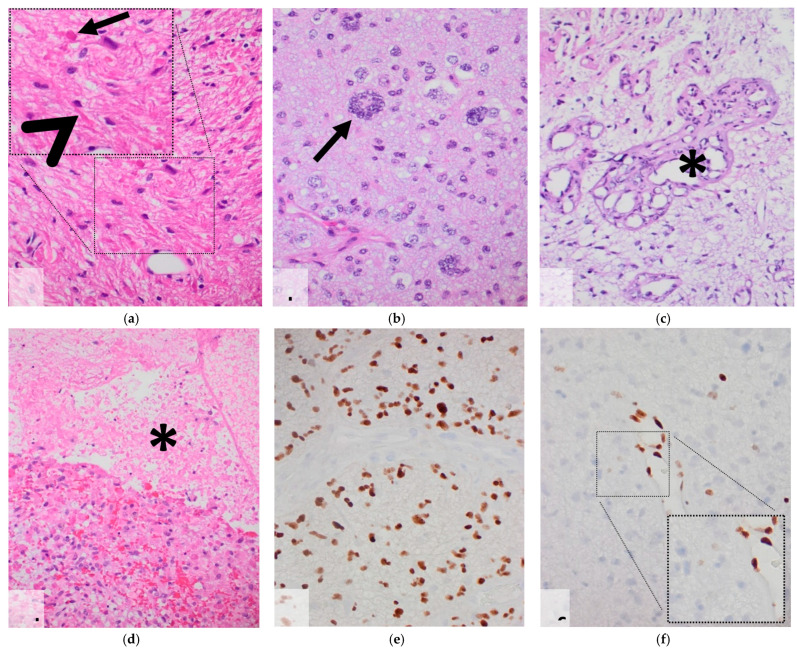
Microscopic pattern of diffuse midline gliomas WHO grade IV H3 K27M-mutated. (**a**) H&E—piloid-like astrocytic tumor cells with Rosenthal fibers (arrow head) and eosinophilic protein droplets (arrow); (**b**) H&E—multinucleated large malignant cells (arrow); (**c**) H&E—multiluminal microvascular proliferates (*); (**d**) H&E—geographic necrosis (*); (**e**) H3 K27M immunohistochemistry—tumor cells expressing mutated H3 K27M protein with negative endothelial cells; (**f**) H3 K27me3 immunohistochemistry—tumor cells showing loss of expression of trimethylated H3 K27 histone protein with positive endothelial cells.

In summary, 39 patients with midline gliomas carried a H3 K27M mutation and matched the inclusion criteria. In this group, 22/39 were male, their age ranging from 1–58 years (mean ± SD: 25.7 ± 16.8), and 11/39 patients received steroid therapy within the last two weeks prior to MR imaging (Table 1). MVP were seen in 24/39 and necrosis in 17/24 tumors (Figure 1). A combination of MVP and necrosis was observed in 18/39. In summary, 24/39 DMGs were DMG-GBMpos and 15/39 were DMG-GBMneg (Table 1).

Fifteen of the 39 DMGs were located in the thalamus, 23/39 in the brainstem and one tumor was located in the vermis of the cerebellum. DMG-GBMneg tumors were located in the thalamus in 5/15 cases and in 10/15 cases in the brainstem. DMG-GBMpos tumors were located in the thalamus in 10/24 cases, in the brainstem in 13/24 cases and in 1 case mainly in the cerebellum. Edema was present in 3/39 cases. Rim enhancement at the peripheral border of the tumor was seen in 17/39 cases, and signs of necrosis were observed in 24/39 cases. Analysis of the enhancement pattern showed strong enhancement in 14/39 cases, intermediate enhancement in 10/39 cases, low enhancement in 10/39 cases and no enhancement in 5/39 cases. Two of the 39 DMGs were multifocal, and 2/39 showed leptomeningeal spread. All DMGs exhibited a local mass effect.

DWI data were available in 31/39 DMG patients. Mean values of rADC measurements were as follows: rADC (mean) = 1.51, rADC (min) = 0.86, rADC (max) = 2.92. Histogram analysis revealed a mean skewness of 1.5 and mean kurtosis of 4.54 for the DMG group. rADC-percentile (10/25/75/90) values were as follows: 1.23/1.31/1.64/1.83. The DMG-GBMneg showed the following mean rADC values: rADC (mean) = 1.59, rADC (min) = 1.0, rADC (max) = 2.66; rADC-percentile (10/25/75/90) values were as follows: 1.33/1.46/1.75/1.94. The group of DMG-GBMpos showed: rADC (mean) = 1.46, rADC (min) = 0.55, rADC (max) = 3.06; rADC-percentile (10/25/75/90) values were as follows: 1.15/1.2/1.55/1.74. Regarding the DMG-subgroups ADC histogram analysis showed the following skewness/kurtosis (DMG-GBMneg vs. DMG-GBMpos): 1.19/4.21 vs. 1.74/4.79. The whole results of the rADC analysis (including confidence intervals (CI)), as well as the rADC histogram analysis, are shown in Table 2 and Table 3.

There was no statistically significant difference between the locations, thalamus vs. brainstem, for any of the imaging characteristics for the whole DMG group. The anatomical distribution between the DMG-GBMneg and DMG-GBMpos tumor was also not statistically different.

### 3.2. MidGBM-H3wt Group

In total, 18 patients with the histopathological diagnosis of midGBM-H3wt met the inclusion criteria (mean 46.2 years, SD 13.2 years; 11 males and 7 females). Twelve of 18 midGBM-H3wt were located in the thalamus; 6/18 tumors were located in the brainstem, and none were located in the cerebellum (Table 2). Steroid therapy was applied to 12/18 patients at the time of imaging.

MRI analysis revealed necrosis in 16/18 patients. Eight of 18 tumors showed strong contrast enhancement; intermediate enhancement was found in 7/18 (39%) and low enhancement in 3/18 cases (17%). In three cases, the contrast enhancement was rated differently between the readers (rated strong or intermediate, respectively), and it was agreed between the readers to put them into the strong enhancement group. Rim enhancement was observed in 12/18 patients (67%). In 14/18 GBM cases, peritumoral edema was identified (78%). Mass effects were seen in all patients, and no intracranial metastasis was observed in the control group. Multifocal lesions were seen in 3/18 patients (16%) (Table 4). Histologically, necrosis was identified in 17/18 patients (94%), and microscopic MVP were found in all 18 tumors (100%).

DWI data were available in 14/18 patients. Mean values of rADC were as follows: rADC (mean) = 1.44, rADC (min) = 0.74, rADC (max) = 2.81. ADC histogram analysis revealed skewness/kurtosis values of 0.86/1.02, and rADC-percentile (10/25/75/90) values were as follows: 1.1/1.23/1.59/1.8. For the results of the rADC histogram analysis, please refer to Table 2 and Table 3.

There was no statistically significant difference between the location, thalamus vs. brainstem, for most of the imaging characteristics. Only low enhancement occurred less frequently in the brainstem (*p* = 0.025).

**Figure 2:**
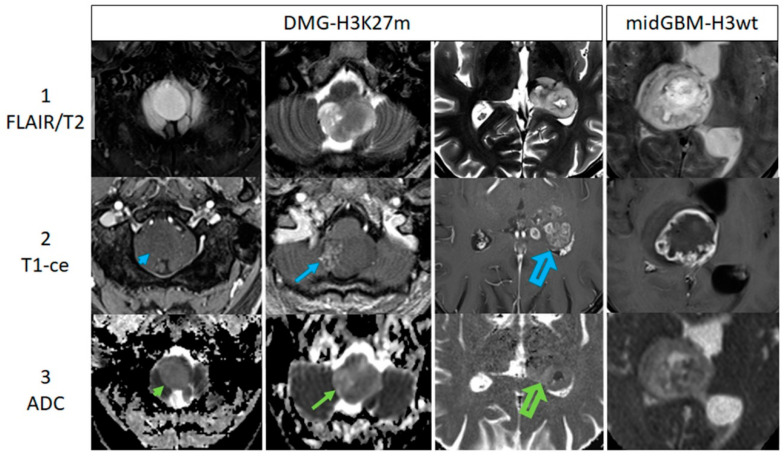
This figure displays examples of MR imaging findings including DWI/ADC. The cases of four patients with midline gliomas are shown in columns from left to right (row 1 presenting FLAIR/T2w-images, row 2 representing ce-T1w images, and row 3 showing ADC maps). The first three columns present DMGs with increasing contrast enhancement from left to right: no enhancement (blue arrowhead), low enhancement (blue arrow) and strong enhancement (blue open arrow). The fourth column shows a case of a midline glioblastoma (midGBM-H3wt). Corresponding ADC-maps are shown in row 3, indicating the variance of inhomogeneity (green arrowhead/arrows pointing at the tumor).

For more details on the MRI signature of both groups, please refer to Table 4. Figure 2 displays examples of MR imaging findings, including DWI/ADC.

### 3.3. Group Comparison

Comparing both groups, patients with midGBM-H3wt were significantly older than those with DMG (*t*-test; *p* = 0.001, 95%-CI: 11.52–29.54). No association was found between sex or tumor location with tumor type, using Fisher’s exact test. MVP (*p* < 0.001) and necrosis (*p* < 0.001) were histologically more frequent in midGBM-H3wt than in DMGs. Peritumoral edema was much less frequent in DMGs (*p* < 0.001) (Table 1 and Table 4). Steroid therapy was more often used in midGBM-H3wt patients compared to DMG patients, and this difference was significant (*p* = 0.018) (Table 1).

The Shapiro–Wilk test did reveal similar variances of rADC between DMG-GBMneg and DMG-GBMpos, as well as midGBM-H3wt (Table 3), while group sizes were almost similar. In this situation, the *t*-test is known to be robust against non-normality, therefore it was continuously used. Relative ADC values (rADC mean, rADC minimum, rADC maximum) did not differ significantly between the groups of DMGs and midGBM-H3wt (*t*-test: *p* = 0.446/0.128/0.663, respectively). Only “rADC min” was associated with the histological features of malignancy, including necrosis and/or microvascular proliferations: In the analysis of the pairs DMG-GBMneg/midGBM-H3wt and DMG-GBMneg/DMG-GBMpos, we observed a significant decrease of rADC (min) in histomorphological high-grade tumors (*p* < 0.001 and *p* = 0.001 respectively). No significant difference could be found regarding rADC mean and rADC maximum in any of the compared pairs (Table 2).

The histogram-based analysis of the rADC data revealed a significant difference for the statistical distribution values kurtosis and skewness between the DMG and midGBM-H3wt groups (*p*-values of 0.016 and 0.002). ROC-/AUC-analysis of these two parameters (DMG vs. midGBM-H3wt) revealed an AUC of 0.733 (*p*-value 0.014, standard error 0.078, CI: 0.58–0.886) for kurtosis and for skewness an AUC of 0.621 (*p*-value 0.199, standard error 0.082, CI: 0.46–0.783). The values for rADC (10) as well as rADC (25) were significantly higher for DMG-GBMneg vs. midGBM-H3wt (*p* = 0.012/0.027), as well as for DMG-GBMneg vs. DMG-GBMpos (*p* = 0.036/0.028) (Table 2). rADC (min) values were also significantly higher when comparing these tumor groups (DMG-GBMneg vs. midGBM-H3wt: *p* < 0.001; DMG-GBMneg vs. DMG-GBMpos: *p* = 0.001). ROC-analysis for rADC (min) revealed the highest AUC of 0.915 (*p* < 0.001, standard error 0.056, CI: 0.805–1) for a comparison of DMG-GBMneg vs. midGBM-H3wt and an AUC of 0.812 (*p* = 0.003, standard error 0.076, CI: 0.663–0.961) for a comparison of DMG-subgroups with and without malignant histological features. An rADC (min) value of 0.89 would lead to a sensitivity of 85% and specificity of 93% for the separation of DMG-GBMneg vs. midGBM-H3wt, while for the separation of DMG-GBMneg vs. DMG-GBMpos, an rADC (min) value of 0.89 would result in a specificity/sensitivity of 61%/92% (alternatively rADC (min) = 0.81: specificity/sensitivity 88%/70%).

Strong contrast enhancement, rim enhancement and necrosis in MRI correlated with the histological features of malignancy but not with the H3 K27M mutation status: a paired comparison of DMG/midGBM-H3wt regarding these three MRI features resulted in *p*-values of 0.262, 0.155 and 0.060, respectively. Six DMGs showed no enhancement but this was not significant over the whole group. On the other hand, the comparison of DMG-GBMneg vs. midGBM-H3wt (regarding strong contrast enhancement, rim enhancement, and signs of necrosis in MRI) resulted in *p*-values of 0.004, <0.001 and <0.001, thus showing statistical significance. All non-enhancing DMGs belonged to the group of DMG-GBMneg. Comparing DMG-GBMneg/DMG-GBMpos (regarding strong contrast enhancement, rim enhancement and signs of necrosis in MRI) the resulting p-values were significant at 0.002, <0.001 and <0.001 (Table 5).

## 4. Discussion

For as long as infratentorial diffuse gliomas have been evaluated solely by histopathology, these tumors were classified and graded like supratentorial tumors [21]. The detection of K27M mutations in histone H3-encoding genes subsequently led to the perception that this group of predominantly astrocytically differentiated tumors represents a distinct tumor type occurring exclusively in the midline structures of the brain [1]. In the course of characterizing such DMGs, it also became clear that the established grading criteria of supratentorial diffuse gliomas was not valid for this tumor type [4,5,6]. However, there are other diffuse gliomas of the midline. These are, for example, gliomas with the typical genetic features of supratentorial gliomas, such as GBM [22] or IDH-mutated diffuse astrocytomas [23]. The latter diffuse gliomas can still be scored with respect to their prognostic value by the established supratentorial grading criteria. Because of their midline location, the resection of diffuse gliomas of the brainstem is often not possible, and stereotactic biopsy is a standard procedure in specialized centers [9,10]. Accordingly, neuroradiological diagnosis in the context of the neurosurgical sampling of such tissue has much greater importance for infratentorial than for supratentorial gliomas. Currently, however, there are only a limited number of studies that have attempted to establish neuroradiological criteria for the identification of DMG, since these tumors occur rather rarely, even in large neurosurgical centers [24,25,26,27]. Accordingly, the few reported case series turned out to be rather small, except for the study by Qiu et al. [26]. The interest in distinguishing DMG from midGBM-H3wt solely on the basis of neuroradiological criteria is high. A particular neuroradiological challenge is the fact that midGBM-H3wt, in most cases, show gross necrosis and MVP, which both induce typical signal alterations in MR imaging. In contrast, a significant number of DMGs show a histopathological DMG-GBMneg pattern [4,5,6], so typical malignancy criteria are more likely to be absent in MR imaging. Modern radiogenomic feature analyses have focused on correlating MRI features with histological characterizations [14] or clinical paths relating to prognosis or therapeutic targets in DMG patients [28]. Wu et al. showed that an analysis combining radiogenomic features and clinical factors yielded better results in predicting H3 K27M status in pediatric high-grade midline gliomas [15]. Since in many clinical situations, a radiogenomic-based analysis of individual cases is not yet available, the analysis of classic MRI features is still important, although previous studies in children have failed to show typical MRI patterns [29]. First-order feature extraction using histogram analysis allows for a more in-depth characterization of MRI signal patterns compared to classic structural MRI features, and several tools are available [20]. This method analyzes images voxel by voxel resulting in a value distribution within the region/volume of interest and thereby also provides information on signal uniformity, heterogeneity and symmetry. An interesting step forward is the application of machine learning (ML) decision-tree models to this type of analysis. Payabvash et al. used this technique based on diffusion histogram analysis as well as structural MRI findings for the differentiation of posterior fossa tumors with promising results, but DMGs were not part of their cohort [17].

By assembling patients from two large neurosurgical centers, we are now in a position to neuroradiologically evaluate a significantly larger number of genetically defined DMGs to compare their MRI features with midGBM-H3wt of the brainstem/thalamus and to analyze such DMGs that would be considered DMG-GBMneg or DMG-GBMpos according to supratentorial histopathological grading criteria. Unfortunately, ML methods require larger datasets due to overfitting problems in smaller sample sizes like ours, which we therefore could not apply.

From our observations, we found the highest percentage of DMGs in the brainstem (23/39), followed by the thalamus (15/39), as was similarly described in previous reports [3,30]. In contrast, Qiu et al. found the majority of DMG tumors in the thalamus (38/66 cases) [26], the location in which we mostly found midGBM-H3wt tumors within our cohort. We also had a single DMG case arising from the cerebellar vermis; this location has previously been mentioned by Hong et al. [19]. However, in our bicenter retrospective cohort we did not observe tumors solely located in other midline sites such as the third ventricle, pineal region or hypothalamus, which were previously described [3,31,32]. Two DMGs were centered in the thalamus but diffusely involved the hypothalamus and medial temporal lobe, as well the pons, with a pattern comparable to the former gliomatosis cerebri. In 2/39 cases, leptomeningeal spread was found; this was less frequent than the reported 20% in the cohort of Seong et al. [33] or the 7/66 cases described by Qiu et al. [26]. We concluded, therefore, that the leptomeningeal spread of DMGs is rare. In our previous work, as well as in this analysis, we encountered a very low frequency (8%) of peritumoral edema, which is similar to Qiu et al. (15%) [26]. Although DMGs are grade IV tumors by definition, often including histomorphological GBM-like features (DMG-GBMpos), this low edema frequency is remarkable when compared to midGBM-H3wt and should not be interpreted as an MRI-sign of a lower grade tumor. The use of corticosteroids cannot account for this difference, since its use occurred even more frequently in the midGBM-H3wt. DMGs can show a varying degree of contrast enhancement, its presence indicating histomorphological GBM-like features. This finding of varying degrees of contrast enhancement between cases is typical and was reported in several studies [26,29]. In our previous study, we found significantly less strong enhancement in the DMG group, which now changed to a non-significant trend of lower frequency in the DMG group compared to the midGBM-H3wt group (38% vs. 56%). This change is likely dependent on the cohort size. Additionally, besides technical aspects of MR image acquisition, corticosteroids also have a known closing effect on the blood brain barrier and, thereby, a reducing effect on the amount of contrast enhancement. For this reason, the use of corticosteroids was included in the RANO criteria for neurooncological response assessment [34]. Corticosteroids were used significantly more frequently within the midGBM-H3wt group, and this might have reduced the frequency of strong enhancements, thereby rendering the group difference non-significant.

DWI techniques allow for the analysis of water mobility and tissue microstructure [35,36]. It can also be used to analyze the microstructure of brain tumors supporting differential diagnosis and grading [37,38,39] and to help evaluate therapy effects [40,41]. ADC values can vary within a single DMG between enhancing and non-enhancing tumor areas, maybe more frequently in cases with ATRX loss [33]. We did not separate enhancing from non-enhancing tumor areas, which might explain the similarity of mean rADC values between the DMG and midGBM-H3wt groups in our study, comparable with the findings of Wu et al. [15]. The use of minimum rADC values is less dependent on these averaging effects and showed significantly higher values in DMG-GBMneg when compared to midGBM-H3wt as well as DMG-GBMpos. This rADC (min) parameter also showed the highest ROC-AUC values of 0.915 and 0.812 when comparing these groups. Similarly, the rADC-percentiles 10, as well as 25, were lower in the tumor groups with histomorphological malignant features (DMG-GBMpos/midGBM-H3wt) when compared to DMG-GBMneg. Therefore, lower minimum rADC values indicate the more complex tissue microstructure, but they cannot designate H3 K27M mutation status. Histogram analysis and the first-order feature extraction is analyzing the differences of signal intensity/value distribution on a voxel by voxel basis and thereby allowing for a better characterization of values than a simple mean value. Our results also indicate the importance of this analysis technique, since the kurtosis and skewness values proved to be significantly different between the DMG and midGBM-H3wt groups only. A higher positive skewness indicates a more right-shifted data distribution, possibly indicating the occurrence of more extreme rADC values on the higher end, since a higher kurtosis also indicates more outliers at the ends of the data distribution. A more positive kurtosis also indicates more data around the mean, while the lower kurtosis values in the midGBM-H3wt group indicate more inhomogeneous rADC values around the mean. rADC (min) values and these first-order characteristics could therefore be implemented into future AI and the so-called radiogenomics analyses. However, biopsy and neuropathological analysis of DMG remains the diagnostic gold standard for the planning of further treatment.

It has been demonstrated that patients with infratentorial diffuse astrocytomas and an IDH mutation have a better prognosis than patients with a DMG [23]. Furthermore, individual cases suggest that patients with infratentorial gliomas and a combined H3F3A K27M and BRAF V600E or FGFR1 mutation have a better prognosis [19,42], while patients with tumors of such a localization and a combined H3 K27M and IDH mutation are more likely to be prognostically related to DMG patients [23,42]. However, since the bioptic sampling of diffuse infratentorial gliomas can be difficult to carry out for individual or institutional reasons, neuroradiological diagnostics remain an important option for the differentiation of tumor types for therapy planning. The differentiation of molecularly defined groups beyond single biomarkers is available by the epigenetic classification of brain tumors [43]. An association of neuroradiological and epigenetic signatures of infratentorial diffuse gliomas is also of particular interest and should be carried out in the future.

The reported median age of patients with a DMG was 5–11 years [1], with a DMG frequency of 50–80% [2], whereas the median age of the patients presented in this work is 25.7 years. However, those published data refer to pediatric cohorts, while we have no current epidemiological data regarding the proportion of DMG patients in mixed pediatric/adult cohorts [44]. We cannot estimate the frequency of DMG in our institutions based on our study design/inclusion criteria. It could only be speculated upon whether the frequency of patients diagnosed with a DMG in the case of a midline tumor decreases noticeably when mixed age groups are analyzed. This could be further analyzed in future studies.

Limitations of this study include a potential bias of the bicenter approach and the nature of its retrospective evaluation. As both centers receive submissions from other hospitals, only the available data were examined; for example, for the majority of patients, no standardized MRI examination of the spinal axis was available, and therefore spinal metastasis could not be completely excluded by this retrospective evaluation. A multivariate analysis of the data is a beneficial tool for interpreting data, but due to the sample size it was not possible to be performed in our study.

The manual placement of ROIs can be prone to error, and automatic segmentation, especially if supported by artificial intelligence (AI), may be preferred. Automatic segmentation is difficult on non-standardized/non-3-dimensional (3D) images, upon which many available AI-algorithms are trained. Therefore, due to the retrospective nature of this study and the inclusion of mostly 2D-images without a standardized imaging protocol coming from different scanning sites, we chose to place ROIs on every slice containing parts of the tumor. For future studies, one should aim at standardized protocols including 3D-datasets as well as quantitative imaging sequences.

## 5. Conclusions

In conclusion, the DMGs in our study cohort had highly variable features on MRI, but exhibited significantly less edema than midGBM-H3wt, even if histomorphological malignancy was present. Leptomeningeal spread was a rare complication of DMGs. On average, DMGs showed lower contrast enhancement compared to midGBM-H3wt. Lower minimum rADC values indicated malignant histomorphological GBM-like features, likely representing a more complex tissue microstructure. First-order feature extraction of ADC values by histogram analysis showed higher kurtosis and skewness values in the DMG group compared to midGBM-H3wt tumors. Therefore, it could be beneficial to include the amount of contrast enhancement, the existence of edema and rADC-minimum values, as well as ADC-based histogram analysis data, as parameters in future machine learning decision trees or pattern analyses.

## Figures and Tables

**Table 1:** Patient characteristics. Patients’ characteristics, steroid therapy at the time of or up to 14 days before imaging and histologic features in relation to tumor type.

Characteristics	DMG *n* (%)	midGBM- H3wt *n* (%)	Total	Statistics DMG vs. midGBM-H3wt	DMG- GBMneg *n* (%)	DMG- GBMpos *n* (%)	Total	Statistics DMG-GBMpos vs. DMG-GBMneg
Number of cases	39 (68)	18 (32)	57		15 (38)	24 (62)	39	
Sex				*p* = 0.781 *				*p* = 1.000 *
Male	22/39 (56)	11/18 (61)	33		8/15 (53)	14/24 (58)	22	
Female	17/39 (44)	7/18 (39)	24		7/15 (47)	10/24 (42)	17	
Age Range	1–58	17–68		***p*< 0.001** ** **95%-CI: 11.52–29.54**	9–54	1–58		*p* = 0.065 ** 95%-CI: 0.65–20.99
Mean ± SD	25.7 ± 16.8	46.2 ± 13.2			32.0 ± 15.7	21.8 ± 16.5		
Steroid therapy	11/39 (28)	12/18 (67)	23	***p*** = 0.018 *	4/15 (27)	7/24 (29)	11	*p* = 1.000 *
Histology								
MVP	24/39 (62) ^#^	18/18 (100)	42	***p*** = 0.001 *	0/15	24/24 (100)	24	
Tumor necrosis	17/39 (44)	17/18 (94)	34	***p*** < 0.001 *	0/15	17/24 (71)	17	

* Using Fischer’s exact test; ** Using *t*-test. ^#^ This complies with the number of DMG-GBMpos. The remaining 15/39 (38%) tumors are DMG-GBMneg. DMG: diffuse midline glioma, H3 K27M-mutant; midGBM-H3wt: glioblastoma of the midline structures, H3 K27M-wildtype, SD: standard deviation; CI: confidence interval; MVP: microvascular proliferations; DMG-GBMpos: diffuse midline glioma, H3 K27M-mutant with glioblastoma histology; DMG-GBMneg: diffuse midline glioma, H3 K27M-mutant without glioblastoma histology. Significant values are shown in bold.

**Table 2:** Distribution of relative ADC histogram parameters and relative mean, minimum and maximum ADC values among the two tumor entities and the DMG-subtypes.

Compared Pairs (n)	rADC (10)	rADC (25)	rADC (75)	rADC (90)	Skewness	Kurtosis	Entropy	rADC (Mean)	rADC (Min)	rADC (Max)
DMG(30)/ midGBM-H3wt (14)	1.23/1.10	1.31/1.23	1.64/1.59	1.83/1.80	1.50/0.85	4.54/1.02	4.27/4.33	1.51/1.43	0.86/0.74	2.92/2.80
*p*-value	0.083	0.445	0.686	0.780	**0.016**	**0.002**	0.737	0.446	0.128	0.633
95%-CI	−0.01–0.26	−013–0.29	−0.18–0.26	−0.21–0.27	0.12–1.17	1.33–5.69	0.46–0.33	−012–0.28	−0.03–0.24	−0.35–0.58
DMG-GBMneg(13)/ midGBM-H3wt (14)	1.33/1.10	1.46/1.23	1.75/1.59	1.94/1.80	1.19/0.85	4.21/1.02	4.39/4.33	1.59/1.44	1.00/0.74	2.66/2.81
*p*-value	**0.012**	**0.027**	0.210	0.298	0.420	0.083	0.838	0.182	**<0.001**	0.589
95%-CI	0.05–0.39	0.02–0.42	−0.09–0.41	−0.13–0.42	−0.53–1.20	−0.47–6.84	−0.48–0.58	−0.07–0.38	0.14–0.36	−0.69–0.40
DMG-GBMneg(13)/ DMG-GBMpos (17)	1.33/1.15	1.46/1.20	1.75/1.55	1.94/1.74	1.19/1.74	4.21/4.79	4.39 /4.17	1.59/1.46	1.00/0.55	2.66/3.06
*p*-value	**0.036**	**0.028**	0.141	0.178	0.253	0.779	0.403	0.279	**0.001**	0.090
95%-CI	−0.01–0.37	−0.00–0.51	−0.07–0.46	−0.09–0.49	−1.53–0.41	−4.81–3.64	−0.29–0.72	−0.11–0.37	0.12–0.39	−0.96–0.073

All numbers shown are mean values. rADC: relative ADC value; min: minimum; max: maximum; DMG: diffuse midline glioma, H3 K27M-mutant; midGBM-H3wt: glioblastoma of the midline structures, H3 K27M-wildtype.; DMG-GBMneg: diffuse midline glioma, H3 K27M-mutant without glioblastoma histology; DMG-GBMpos: diffuse midline glioma, H3 K27M-mutant with glioblastoma histology; CI: confidence interval regarding differences of mean values in each compaired pair. Significant values are shown in bold (*t*-test).

**Table 3:** Results of histogram as well as whole-tumor-ROI (r)ADC analysis for the different tumor groups. Normal distribution analysis using the Shapiro–Wilk test, result is indicated by the *p*-value.

Tumor Groups (n)	rADC (10)	rADC (25)	rADC (75)	rADC (90)	Skewness	Kurtosis	Entropy	rADC (Mean)	rADC (Min)	rADC (Max)
DMG (30 *)										
*p*-value	0.342	0.105	0.225	0.464	**0.045**	**<0.001**	0.862	0.425	**0.044**	0.107
Mean ± SD	1.23 ± 0.27	1.31 ± 0.36	1.64 ± 0.36	1.83 ± 0.39	1.50 ± 1.29	4.54 ± 5.51	4.27 ± 0.67	1.51 ± 0.33	0.85 ± 0.23	2.92 ± 0.71
95%-CI **	1.12–1.33	1.18–1.45	1.50–1.77	1.68–1.98	1.01–1.99	2.48–6.60	4.01–4.52	1.39–1.63	0.76–0.94	2.65–3.18
midGBM-H3wt (14)										
*p*-value	0.562	0.908	0.352	0.293	0.868	**0.011**	0.620	0.492	**0.029**	0.928
Mean ± SD	1.10 ± 0.17	1.23 ± 0.21	1.59 ± 0.27	1.80 ± 0.32	0.85 ± 0.37	1.02 ± 1.38	4.33 ± 0.44	1.43 ± 0.27	0.74 ± 0.15	2.80 ± 0.74
95%-CI **	1.00–1.21	1.11–1.35	1.43–1.75	1.61–1.98	0.63–1.07	0.22–1.83	4.08–4.59	1.28–1.59	0.65–0.83	2.38–3.23
DMG-GBMneg (13)										
*p*-value	0.393	0.200	0.097	0.052	**0.017**	**<0.001**	0.885	0.126	0.811	0.103
Mean ± SD	1.33 ± 0.24	1.46 ± 0.28	1.75 ± 0.35	1.94 ± 0.38	1.19 ± 1.40	4.21 ± 5.96	4.39 ± 0.81	1.59 ± 0.31	1.00 ± 0.12	2.66 ± 0.62
95%-CI **	1.18–1.48	1.28–1.63	1.53–1.97	1.71–2.18	0.33–2.04	0.60–7.81	3.89–4.88	1.40–1.78	0.92–1.07	2.28–3.04
DMG-GBMpos (17 *)										
*p*-value	0.485	0.371	0.792	0.955	0.731	**0.008**	0.548	0.926	0.650	0.063
Mean ± SD	1.15 ± 0.27	1.20 ± 0.38	1.55 ± 0.35	1.74 ± 0.38	1.74 ± 1.19	4.79 ± 5.31	4.17 ± 0.55	1.46 ± 0.34	0.74 ± 0.24	3.10 ± 0.73
95%-CI **	1.01–1.29	1.00–1.40	1.37–1.73	1.54–1.94	1.13–2.36	2.06–7.52	3.89–4.46	1.29–1.63	0.62–0.86	2.74–3.47

* rADC (mean/min/max) data were available for 31 DMG and 18 DMG-GBMpos, respectively. ** 95% confidence interval of the mean value. rADC: relative ADC value; min: minimum; max: maximum; DMG: diffuse midline glioma, H3 K27M-mutant; midGBM-H3wt: glioblastoma of the midline structures, H3 K27M-wildtype.; DMG-GBMneg: diffuse midline glioma, H3 K27M-mutant without glioblastoma histology; DMG-GBMpos: diffuse midline glioma, H3 K27M-mutant with glioblastoma histology; CI: confidence interval. Significant *p*-values are shown in bold.

**Table 4:** MRI findings and their distribution in DMG and midGBM-H3wt cohorts, including correlation analysis data.

Imaging Features		DMG n (%)	midGBM-H3wt n (%)	*p*-Value
Location	thalamus	15/39 (38)	12/18 (67)	0.086
	brainstem	23/39 (59)	6/18 (33)	0.092
	cerebellum	1/39 (3)	0/18	0.536
Edema present		3/39 (8)	14/18 (78)	**<0.001**
Rim enhancement		17/39 (44)	12/18 (67)	0.155
Tumor necrosis		24/39 (62)	16/18 (89)	0.060
Strong enhancement		15/39 (38)	10/18 (56)	0.262
Low enhancement		15/39 (38)	3/18 (17)	0.132
Mass effect		39/39 (100)	18/18 (100)	
Multifocal lesions		2/39 (5%)	3/18 (17)	0.312
Intracranial metastasis		2/39 (5%)	0/18	1.000

DMG: diffuse midline glioma, H3 K27M-mutant; midGBM-H3wt: glioblastoma of the midline structures, H3 K27M-wildtype. Significant values are shown in bold.

**Table 5:** Comparison of different tumor groups in terms of strong enhancement, rim enhancement and necrosis in MRI considering histological features of malignancy (microvascular proliferations, microscopic necrosis).

Compared Pairs (n)	Strong Enhancement %/%	Rim Enhancement %/%	Necrosis in MRI %/%
DMG (39)/midGBM-H3wt (18)	38%/56%	44%/67%	62%/89%
*p*-value	0.262	0.155	0.060
DMG-GBMneg (15)/midGBM-H3wt (18)	7%/56%	0/67%	7%/89%
*p*-value	**0.004**	**<0.001**	**<0.001**
DMG-GBMneg (15)/DMG-GBMpos (24)	7%/58%		
*p*-value	**0.002**		

DMG: diffuse midline glioma, H3 K27M-mutant; midGBM-H3wt: glioblastoma of the midline structures, H3 K27M-wildtype.; DMG-GBMneg: diffuse midline glioma, H3 K27M-mutant without glioblastoma histology; DMG-GBMpos: diffuse midline glioma, H3 K27M-mutant with glioblastoma histology. Significant *p*-values are shown in bold.

## Data Availability

The data presented in this study are available on request from the corresponding authors.
